# Early administration of IL-6RA does not prevent radiation-induced lung injury in mice

**DOI:** 10.1186/1748-717X-5-26

**Published:** 2010-04-07

**Authors:** Toshiyuki Ogata, Hideya Yamazaki, Teruki Teshima, Ayaka Kihara, Yuko Suzumoto, Takehiro Inoue, Norihiro Nishimoto, Nariaki Matsuura

**Affiliations:** 1 Department of Radiation Oncology, Osaka University Graduate School of Medicine, 2-2 Yamadaoka, Suita, Osaka, Japan; 2 Department of Radiology, Graduate School of Medical Science, Kyoto Prefectural University of Medicine, Kajiicho, Kawaramachi Hirokoji, Kamigyo-ku, Kyoto, Japan; 3 Medical Physics & Engineering, Osaka University Graduate School of Medicine, 2-2 Yamadaoka, Suita, Osaka, Japan; 4 Laboratory of Immune Regulation, Wakayama Medical University, Saito-asagi, Ibaraki, Osaka, Japan; 5 Functional Diagnostic Science, Osaka University Graduate School of Medicine, 2-2 Yamadaoka, Suita, Osaka, Japan

## Abstract

**Background:**

Radiation pneumonia and subsequent radiation lung fibrosis are major dose-limiting complications for patients undergoing thoracic radiotherapy. Interleukin-6 (IL-6) is a pleiotropic cytokine and plays important roles in the regulation of immune response and inflammation. The purpose of this study was to investigate whether anti-IL-6 monoclonal receptor antibody (IL-6RA) could ameliorate radiation-induced lung injury in mice.

**Methods:**

BALB/cAnNCrj mice having received thoracic irradiation of 21 Gy were injected intraperitoneally with IL-6RA (MR16-1) or control rat IgG twice, immediately and seven days after irradiation. Enzyme-linked immunosorbent assay was used to examine the plasma level of IL-6 and serum amyloid A (SAA). Lung injury was assessed by histological staining with haematoxylin and eosin or Azan, measuring lung weight, and hydroxyproline.

**Results:**

The mice treated with IL-6RA did not survive significantly longer than the rat IgG control. We observed marked up-regulation of IL-6 in mice treated with IL-6RA 150 days after irradiation, whereas IL-6RA temporarily suppressed early radiation-induced increase in the IL-6 release level. Histopathologic assessment showed no differences in lung section or lung weight between mice treated with IL-6RA and control.

**Conclusions:**

Our findings suggest that early treatment with IL-6RA after irradiation alone does not protect against radiation-induced lung injury.

## Background

Radiation pneumonia, an interstitial pulmonary inflammation, and subsequent radiation lung fibrosis are significant dose-limiting complications and may threaten quality of life for patients receiving radiation to the thorax. Radiation pneumonia and/or pulmonary fibrosis occur in approximately 10-20% of patients treated with thoracic radiotherapy [1]. The incidence of radiation-induced lung toxicity has increased in recent years due to more aggressive therapies such as combined chemoradiotherapy [2]. Clinical symptoms range from cough, fever, and shortness of breath to death from respiratory failure. At the cellular and tissue level, radiation pneumonia presents as an edema of the interstitial space, infiltration of inflammatory cells, and thickening of the alveolar septa.

Although the molecular mechanism for radiation pneumonia is complex and obscure, involvement of proinflammatory cytokines, chemokines, and cell adhesion molecules has been implicated [3,4]. Many investigators have shown that cytokines play essential roles in the pathogenesis of radiation pneumonia [5,6]. Interleukin-6 (IL-6), which was originally identified as a B-cell differentiation factor [7], is now known to be a multifunctional cytokine that regulates acute phase response, immune response, and inflammation [8,9]. IL-6 is produced by a variety of cells such as T cells, B cells, monocytes, macrophages, fibroblasts, endothelial cells, and several tumor cells [10]. Clinical as well as experimental findings have suggested the involvement of IL-6 as a pro-inflammatory cytokine in radiation pneumonia [11-14]. Indeed, up-regulation of IL-6 production has been observed in human and animals with radiation pneumonia.

In this study, we examined the effect of anti-IL-6 monoclonal receptor antibody (IL-6RA) on radiation-induced lung injury in mice having received lethal irradiation of the whole thorax. We investigated whether IL-6RA treatment would offer the promise of a new pharmacologic intervention strategy for ameliorating radiation-induced lung injury.

## Methods

### Mice and irradiation

Eight-week-old specific pathogen-free female BALB/cAnNCrj mice were obtained from Charles River 1-2 weeks before testing. Mice were maintained according to the institutional animal care use committee guidelines. The mice were anesthetized by i.p. injection of pentobarbital (40 mg/kg) immediately before irradiation. The whole thorax was irradiated by 4 MV X-ray from the linear accelerator (Mitsubishi, EXL-6SP) at a dose of 21 Gy with a delivered dose rate of approximately 1.8 Gy/min and a 1.0-cm bolus material on the surface. The field was 2.5 cm in length in the cephalic-tail direction. Survival and body mass were monitored in IL-6RA treatment (n = 7) or control (n = 8) mice for 200 days after 21 Gy irradiation. For other assays, the animals were sacrificed at a predetermined time of 50, 100, and 150 days after irradiation (n = 5 per group). Any mouse showing signs of distress, including lethargy, hunched back, or increased breathing frequency was sacrificed by overdose of pentobarbital.

### Injection of IL-6RA(MR16-1)

Basic characterizations of the rat anti-mouse IL-6 receptor monoclonal antibody, MR16-1, have been detailed in previously published reports [15]. Immediately and 1 week after irradiation, mice were intraperitoneally administered with a single doses of MR16-1 (8 mg/kg body weight) or with the same volume and concentration of purified rat IgG (ICN Biomedicals, Inc).

### Circulating IL-6 and SAA analysis

Blood samples were collected via cardiac puncture at the time of euthanasia, and plasma was obtained after microcentrifugation at 4,000 g for 5 min. Plasma concentrations of IL-6 (Pierce Endogen Co. Ltd) and serum amyloid A (SAA) (Biosource) were measured by commercial enzyme-linked immunosorbent assay (ELISA) kits according to the instructions of the manufacturer.

### Lung hydroxyproline determination

Collagen deposition was estimated by determining the hydroxyproline content of the left lung. Samples were hydrolyzed with 6 N HCl at 105°C for 18 h. The samples were resuspended in 2 ml of deionized water and 1 ml of chloramine T dissolved in 5 mol/L sodium acetate/10% isopropanol. Next, 0.5 ml of Ehrlich's reagents were added, mixed, and incubated at 65°C for 10 min. The absorbance of the solutions at 562 nm was determined. The hydroxyproline values of the samples were calculated according to comparing to the standard curve. Data were corrected for total left lung wet weight.

### Histologic analysis

For histological examination, the lung tissue was fixed in 10% neutral-buffered formalin solution, embedded in paraffin wax, sectioned (5 μm thickness), and stained with haematoxylin and eosin (H&E) or Azan. For H&E staining, we evaluated an edema of the interstitial space, infiltration of inflammatory cells, thickening of the alveolar septa, and vessel thrombosis. The amount of pulmonary interstitial collagen was determined by Azan staining. The area stained blue (collagen fibers) was evaluated under a microscope.

### Statistical analysis

The statistical significance was tested by means of the Kaplan-Meier method and the log-rank test for survival analysis. Comparison of the other assays between two groups was performed using non-parametric the Mann-Whitney U-test because of small number. A p-value of less than 0.05 was considered to be statistically significant.

## Results

### Survival and body weight

**Figure 1:**
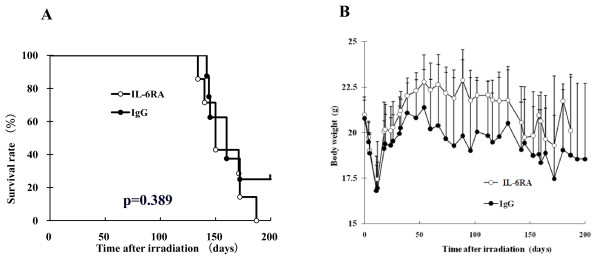
**Kaplan-Meier analysis of survival curves and body weights for IL-6RA- (white circle) and IgG control (black circle)-injected mice**. (A) Survival curves. The *p *value was calculated with the log-rank test. Kaplan-Meier plots were calculated from data for 7 IL-6RA-treated mice and 8 nontreated mice. (B) Body weights. Data are presented as means + SD.

We first evaluated whether IL-6RA administration leads to increased survival of mice exposed to lethal thoracic irradiation and found that the mice treated with IL-6RA did not survive significantly longer than the rat IgG control (Figure 1A). Growth expressed as total body weight is presented graphically in Figure 1B, showing that the mice treated with IL-6RA tended to weigh more than the rat IgG control mice.

### IL-6 and SAA concentrations

**Figure 2:**
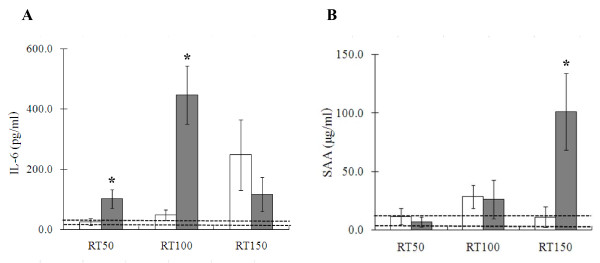
**Effects of IL-6RA treatment in lungs of lethally irradiated IL-6RA (white square) and IgG control (black square) mice on radiation-induced increases in IL-6 (A) and SAA (B) production**. Each bar represents the mean ± SD (n = 5 per group). Asterisks indicate statistical significance. Dotted line represents each from average value of non-irradiated mice + SD to average value of them -SD.

Protein levels of IL-6 and SAA, one of the major acute-phase proteins in mammals, were determined by means of specific ELISA in plasma from irradiated mice (Figure 2). The mice treated with IL-6RA showed a marked reduction of the radiation-induced increase in IL-6 proteins in plasma as compared with the control group 50 days (25.1 ± 10.5 versus 101.6 ± 31.2 pg/ml, p < 0.05) and 100 days (48.5 ± 17.2 versus 446.3 ± 96.9 pg/ml, p < 0.05) after irradiation (Figure 2A). After 150 days, however, a marked, but not statistically significant, up-regulation in IL-6 was observed in mice treated with IL-6RA in comparison with the rat IgG group (247.6 ± 116.6 versus 116.9 ± 56.4 pg/ml). As shown in Figure 2B, administration of IL-6RA significantly suppressed the radiation-induced increase in SAA proteins in plasma compared with the control group after 150 days (10.9 ± 8.8 versus 101.1 ± 32.7 μg/ml, p < 0.05). Non-irradiated mice without any antibody in a previous study showed low IL-6 and SAA protein levels in plasma (25.2 ± 7.7 pg/ml and 8.2 ± 4.4 μg/ml, respectively).

### Wet lung weights and hydroxyproline content

**Figure 3:**
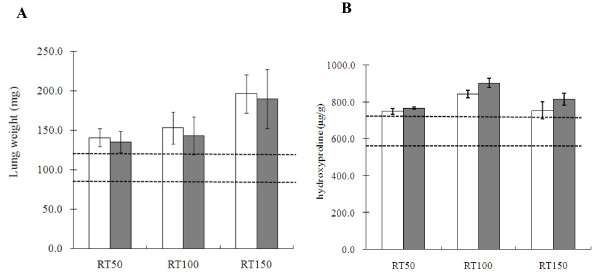
**Effects of IL-6RA treatment on radiation-induced lung injury indices**. (A) Lung weight after 21 Gy of single irradiation to whole thorax with (white square) or without (black square) IL-6RA treatment. (B) Hydroxyproline levels in lung homogenates obtained from IL-6RA injected mice (white square) or control (black square). Data obtained from 5 animals in each group are presented as means ± SD. Dotted line shows each from mean value of non-irradiated mice + SD to mean value of them -SD.

Lung weight was measured to assess pulmonary edema and consolidation (Figure 3A) and after lung irradiation a gradual, time-dependent increase in lung weight was observed in the IL-6RA group as compared with the control group. However, the difference in lung weight between the two groups did not become significant at any time after irradiation.

Lung hydroxyproline measurements were analyzed for the quantification of collagen deposition (Figure 3B) and no statistically significant difference in hydroxyprolin content between the two groups was detected after irradiation.

### Histologic analysis

**Figure 4:**
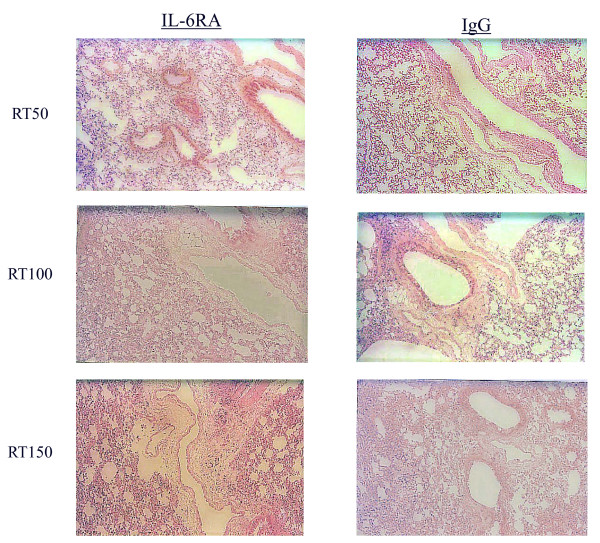
**Histological analyses using H&E staining for irradiated murine lung tissue**.

**Figure 5:**
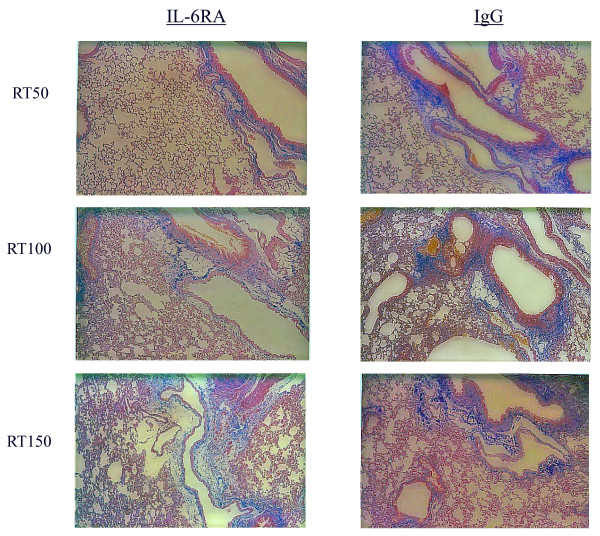
**Histological analyses using Azan staining for irradiated murine lung tissue**.

The time course of H&E and Azan staining in the lung tissue after irradiation is shown in Figures 4 and 5, demonstrating that the extent and severity of lung damage was not significantly reduced in the IL-6RA group in comparison with the control group.

## Discussion

Radiation pneumonia and subsequent radiation lung fibrosis are major dose-limiting complications for patients undergoing thoracic radiotherapy. Recent research findings support the existence of a mechanism of cellular interaction between lung parenchymal cells and circulating immune cells mediated through various cytokines such as proinflammatory cytokines, chemokines, adhesion molecules, and profibrotic cytokines [3,4]. Since IL-6 is a pleiotropic cytokine that plays important roles in the regulation of immune response and inflammation, IL-6 receptor monoclonal antibody treatment has been identified as a promising treatment for Castleman's disease, rheumatoid arthritis, juvenile idiopathic arthritis, and Crohn's disease [16-19]. IL-6 has also been implicated in the pathogenesis of radiation pneumonia [11-14] and is synthesized by type II pneumocytes, alveolar macrophages, T lymphocytes, and lung fibroblasts [5]. We therefore hypothesized that blockage of the IL-6 signaling pathways may offer an attractive therapeutic target for the amelioration of radiation-induced lung injury.

IL-6RA treatment was found to inhibit a radiation-induced increase in IL-6 proteins 50 and 100 days after irradiation. Moreover, after 150 days IL-6RA significantly suppressed a radiation-induced increase in inflammatory marker SAA proteins in plasma as compared with the control group. Acute phase protein SAA is known as a sensitive systemic marker of inflammation and tissue damage [20]. Further, IL-6 plays an important role in the synergistic induction of the SAA gene and the anti-IL-6 receptor monoclonal antibody inhibits the synergistic induction of SAA [21]. These findings indicate that IL-6RA has some beneficial effects. In our study, however, no significant difference was observed in severity of lung damage or length of survival between IL-6RA treated mice and control.

One possible explanation for this finding is that long-term continuous administration of IL-6RA may be necessary for reducing lung toxicity. Rube *et al*. showed that radiation-induced release of IL-6 in the bronchiolar epithelium of C57BL/6J mice was detected a few hours and several weeks after irradiation (peak at 8 weeks) [14]. Radiation-induced lung injury is a chronic phenomenon mediated by various cells such as inflammatory cells responding to the release or activation of downstream cytokines, growth factors, or chemokines. Anscher *et al*. reported long-term (6-month) administration of the small molecule inhibitor of TGF-beta was more effective in reducing radiation-induced lung toxicity than short-term (3-week) administration [22]. Long-term IL-6RA treatment following irradiation may therefore ameliorate radiation-induced lung injury. Because we were concerned that repeated treatment with rat antibody would result in the production of mouse anti-rat antibodies against the rat antibody, we could not administer IL-6RA more than twice. In a previous pilot study we examined plasma IL-6 levels in mice having received only radiation of 21 Gy and confirmed that radiation-induced IL-6 production in Balb/c mice reached a peak 1 week after irradiation (data not shown). The reason for the choice of this time point was that we wanted to inhibit acute interstitial inflammation.

A marked up-regulation of IL-6 was observed in mice treated with IL-6RA in comparison with the rat IgG group 150 days after irradiation. This result may be explained by the fact that the time until the peak concentration of IL-6 in IL-6RA-treated mice had changed due to the inhibition of autocrine production of IL-6. Rube *et al*. reported radiation-induced IL-6 production in C57BL/6J mice reached two peaks a few hours and 8 weeks after irradiation [14]. However, marked mouse strain differences in cytokine levels and patterns may occur such as in the histological configuration of radiation-induced lung injury [23]. BALB/c mice were chosen for this study because this strain is known to possess comparable radiosensitity [24]. We hypothesized that use of a different mice strain might cause changes in the pathogenesis of radiation pneumonia and thus alter the survival of mice receiving lethally irradiation of the whole thorax. A significant increase in SAA was observed in mice treated with IgG in comparison with the IL-6RA group 150 days after irradiation, whereas the mice treated with IL-6RA showed a significant induction of IL-6 in comparison with the rat IgG group. This result may be explained by the fact that signal transduction of IL-1 or TNF-alpha is more strongly involved in the regulation of SAA production than that of IL-6 [25]. These findings suggest that elevation of IL-6 was not strongly implicated in the pathogenesis of radiation pneumonia but was rather derived from the development of radiation pneumonia. Although a limitation of our study is that a relatively small number of mice were used, it is clear from these results that further intensive study is warranted.

There is concern that intervention to prevent radiation-induced toxicity may also serve to protect cancer because clinical investigations found increased serum IL-6 levels in cancer patients [26]. Aberrant production and signaling of circulating IL-6 has been implicated in tumor generation and poor disease outcome in various cancers [27]. Since blockade of IL-6 signaling by shRNA inhibits lung adenocarinoma cell growth [28], IL-6RA treatment may inhibit radiation-induced lung toxicity as well as tumor proliferation.

## Conclusions

To summarize, the findings presented here suggest that early intervention using IL-6RA could not ameliorate radiation-induced lung injury. Additional work is needed to determine the optimal timing and duration for therapy using this approach to the prevention of lung injury after radiation therapy. Despite the negative findings of our study, further intensive studies are needed into strategies for inhibition of cytokine signaling as a way to ameliorating lung toxicity from radiotherapy.

## Competing interests

The authors declare that they have no competing interests.

## Authors' contributions

TO carried out the animal experiments and drafted the manuscript. AK and YS performed the animal experiments. TT and IT participated in the statistical analysis. HY, NN, and MN conceived of the study, and participated in its design and coordination and helped to draft the manuscript. All authors read and approved the final manuscript.
